# Protecting the Cancer Susceptibility Curve

**DOI:** 10.1289/ehp.1307084

**Published:** 2013-08-01

**Authors:** Adam M. Finkel

**Affiliations:** Penn Program on Regulation, University of Pennsylvania Law School, Philadelphia, Pennsylvania, E-mail: afinkel@law.upenn.edu

I was dismayed, but not surprised, to read Charles Schmidt’s excellent article on the importance of considering human variation in susceptibility when regulating environmental health hazards, and to find the word and concept “cancer” mentioned not even once (Schmidt 2013). After all, for 30 years the U.S. Environmental Protection Agency (EPA) has agonized over whether a factor of 3 versus 10 (or less, or more) is adequate to protect the millions of humans who are more susceptible to non-cancer disease processes than the average person, but all the while making no similar effort to account for the substantial and perhaps greater variation in how individuals respond to carcinogenic stimuli.

We estimate carcinogenic potency by fitting a dose–response function to tumor incidence data from groups of 50 inbred rodents—or from somewhat larger groups of working-age people—and simply pronounce the group potency estimate relevant for hundreds of millions of outbred humans, who among them exhibit every conceivable genetic polymorphism, co-exposure, psychological stress factor, and disease status that is deliberately engineered out of the laboratory or attenuated in the epidemiological raw material. When we conclude summarily that an allowable exposure would yield a precise point estimate of, for example, no more than 1-in-10,000 excess cancer risk, we know that many individuals will instead face a risk of 1-in-1,000, or even higher. However, we hide that knowledge from potentially affected persons and provide no interindividual adjustment on the cancer side of environmental health as we do for other types of adverse health effects.

Two successive committees of the National Academy of Sciences have urged the U.S. EPA to abandon the fiction that its cancer risk estimates are “plausible upper bounds,” either of individual risk or of population risk [National Research Council (NRC) 1994, 2009]. (I was a member of both of these large consensus committees.) The more recent report (NRC 2009) went further and also recommended that the agencies “immediately” adjust their individual risk estimates for carcinogens upward by a factor of between 10- and 50-fold, based on evidence that at least 5% of humans are at least this susceptible compared with the average person, and that they adjust the corresponding population risk estimates (body counts) upward by a factor of roughly 7-fold. [For a mathematical derivation of how ignoring variability leads to underestimation of the population mean, see pp. 168–169 of NRC (2009).] The U.S. EPA’s instinct has instead been to offer post hoc rationalizations for ignoring variation in cancer susceptibility; none holds up to scrutiny.

For the first decade after *Science and Judgment in Risk Assessment* (NRC 1994), the U.S. EPA’s various draft cancer guidelines contained this explanation:

The EPA has considered [the NAS recommendation] but decided not to adopt a quantitative default factor for human differences in susceptibility [to cancer] when a linear extrapolation is used. In general, the EPA believes that the linear extrapolation is sufficiently conservative to protect public health. (U.S. EPA 1996)

Obviously, linear extrapolation is not conservative at all if it is actually the correct model for the substance at issue; even if the U.S. EPA does overestimate a dose–response slope, it is unlikely that it does so by a compensating amount sufficient to cause the “two wrongs to equal one right” (Hattis and Goble 1996).

When the U.S. EPA finalized the cancer guidelines in 2005, it garbled the logic further, claiming that linear extrapolation

is thought to be public-health protective at low doses for the range of human variation … although it may not completely be so if pre-existing disease or genetic constitution place a percentage of the population at greater risk from exposure to carcinogens. (U.S. EPA 2005)

It is unclear who “thinks” this, or why, but the entire and unarguable point is that “pre-existing disease and genetic constitution” absolutely do—not “if”—place some individuals at greater risk (Svensson et al. 2006).

More recently, the U.S. EPA has simply begun asserting that its procedures have evolved to consider human variation in cancer risk (see question 6 in U.S. EPA 2011), but these amount to one trivial adjustment and one empty promise. In particular, the upward potency adjustment for children (10-fold for the first 2 years of life, 3-fold for the next 14 years) amounts to only a factor of 1.7 over the life span, which provides scant protection for susceptible adults exposed throughout life, and no additional protection for adults whose exposures begin or increase after 16 years of age. In addition, the U.S. EPA may incorporate chemical-specific data on susceptibility when they exist, but this formulation is the opposite of the sensible default the NAS has twice called for, because it treats human variation as nonexistent until proven otherwise. Having thus been awarded a strong disincentive, the regulated community or academic researchers are not likely to rush to perform the research needed to overturn this untenable but chemical-friendly default.

Why might the U.S. EPA and its stakeholders be spending so much effort refining allometric scaling procedures, dialing back the estimation of exposure to the maximally exposed individual, and positing sophisticated nonlinear modes of action, while continuing to make the unscientific assertion that we are all equally susceptible to carcinogenesis? I observe that the first three improvements tend to result in lower estimated risk and less environmental protection, whereas shining a light on human variation in cancer susceptibility would tend to have the opposite effect on risk estimates.

We should be advancing sound science along all fronts, not only the areas that support one type of policy preference.
